# Effects of integrative neuromuscular training intervention on physical performance in elite female table tennis players: A randomized controlled trial

**DOI:** 10.1371/journal.pone.0262775

**Published:** 2022-01-20

**Authors:** Jinfeng Xiong, Shangxiao Li, Aibin Cao, Lei Qian, Bo Peng, Dandan Xiao

**Affiliations:** 1 Research Center for Sports Psychology and Biomechanics, China Institute of Sport Science, Beijing, China; 2 School of Physical Education, Shanxi University, Taiyuan, Shanxi, China; 3 School of Sciences, Xi’an Technological University, Xi’an, Shaanxi, China; 4 Department of Sports, China University of Political Science and Law, Beijing, China; Universidade Federal do Rio Grande do Sul, BRAZIL

## Abstract

**Objectives:**

To investigate the effects of integrative neuromuscular training (INT) on physical performance in elite female table tennis players.

**Methods:**

Twenty-four Chinese elite female table tennis players were randomized into either INT (n = 12) group or control group (CON; n = 12). INT group performed four INT sessions every week for 8 weeks, while CON group performed traditional physical fitness training. One repetition maximal (1RM), vertical jump, Y balance test and 30-meter sprinting performance were tested before and after intervention.

**Results:**

No between groups differences were detected for any tests before intervention. Significant group by time (before or after intervention) interaction effects were observed in 1RM, vertical jump, bilateral lower limb reaching distance at posteromedial and posterolateral directions, and right leg at the anterior direction for the Y balance test (all *p* < 0.05), but not for the left leg at the anterior direction or the 30-meter sprinting performance (both *p* > 0.05). Post-hoc analysis for measurements with significant interactions revealed that all significant changes were at the ING group (all *p* < 0.05), while no changes for the CON group were observed (all *p >* 0.05).

**Conclusion:**

Eight weeks INT significantly improved strength, power and balance in Chinese elite female table tennis players. Adopting INT in table tennis players may improve their physical performance and lead to better sports performance.

## Introduction

Table tennis is a popular sport with over 300 million people actively participate [1, 2]. There is a great physical demand for professional table tennis players. Players must be able to keep high level performance for over ten days in order to achieve excellent results when playing major tournaments. Therefore, to be able to consistently perform well throughout a tournament, a good table tennis player must possess not only good technical skills, but also great physical fitness level [3].

For physical fitness training programs, strength, power [4], coordination [5] and specific running speed [6] should be considered as key components when designed for table tennis players, as these factors have been shown to have direct link with sports performance. In general, muscle strength, and coordination in table tennis players are related to their rankings [7]. Specifically, those with better strength, power and coordination tend to complete the stroke movement with better quality [8]. In addition, for the most common movements in table tennis, such as swinging, sprinting and turning, a high level of coordination is required to integrate different movement components together [9, 10]. Therefore, training programs that incorporate all abovementioned aspects of physical fitness could be beneficial for sports performance for table tennis players.

However, fitness training programs adopted by table tennis players usually focused on specific aspect of physical fitness but not comprehensive. It’s not uncommon to see training programs for table tennis players to emphasize primarily on strength and speed while little effort is made to simultaneously improve power or coordination. There is a need for a more efficient training modality which can improve multiple aspects of physical fitness within a relative short training period for professional table tennis players. Integrative neuromuscular training (INT), an emerging training modality that combines strength, speed, ultra-length and balance training together with typical functional training [11–15], may be used for this specific purpose. INT has been shown to be able to improve multiple aspects of physical performance for players from many sports like basketball and soccer, including strength [16], power [17], balance [18] and speed [17]. Yet, to the best of our knowledge, no studies have evaluated the effects of INT on physical performance in elite professional table tennis players, who present specific physical demands due to the characteristics of the sport.

Therefore, the purpose of this study is to fill this gap in literature by investigating the effects of INT intervention on physical performance in the Chinese women’s table tennis players. We hypothesized that an 8-week INT intervention protocol can significantly improve strength (as measured using 1 repetition maximal; 1RM), power (as measured by vertical jump test), coordination (as measured using Y balance test), and speed (as measured using 30 meters sprinting test) based on literature reporting effects of INT on other sports.

## Methods

### Ethics statement

This study was approved by the Institutional Review Board at China Institute of Sport Science under approval number CISSIRD-20190104. It was registered at the Chinese Clinical Trial Registry with the registration number ChiCTR2100045673. The protocol and CONSORT checklist and flow chart are available as S1 Checklist.

### Participants

**Fig 1:**
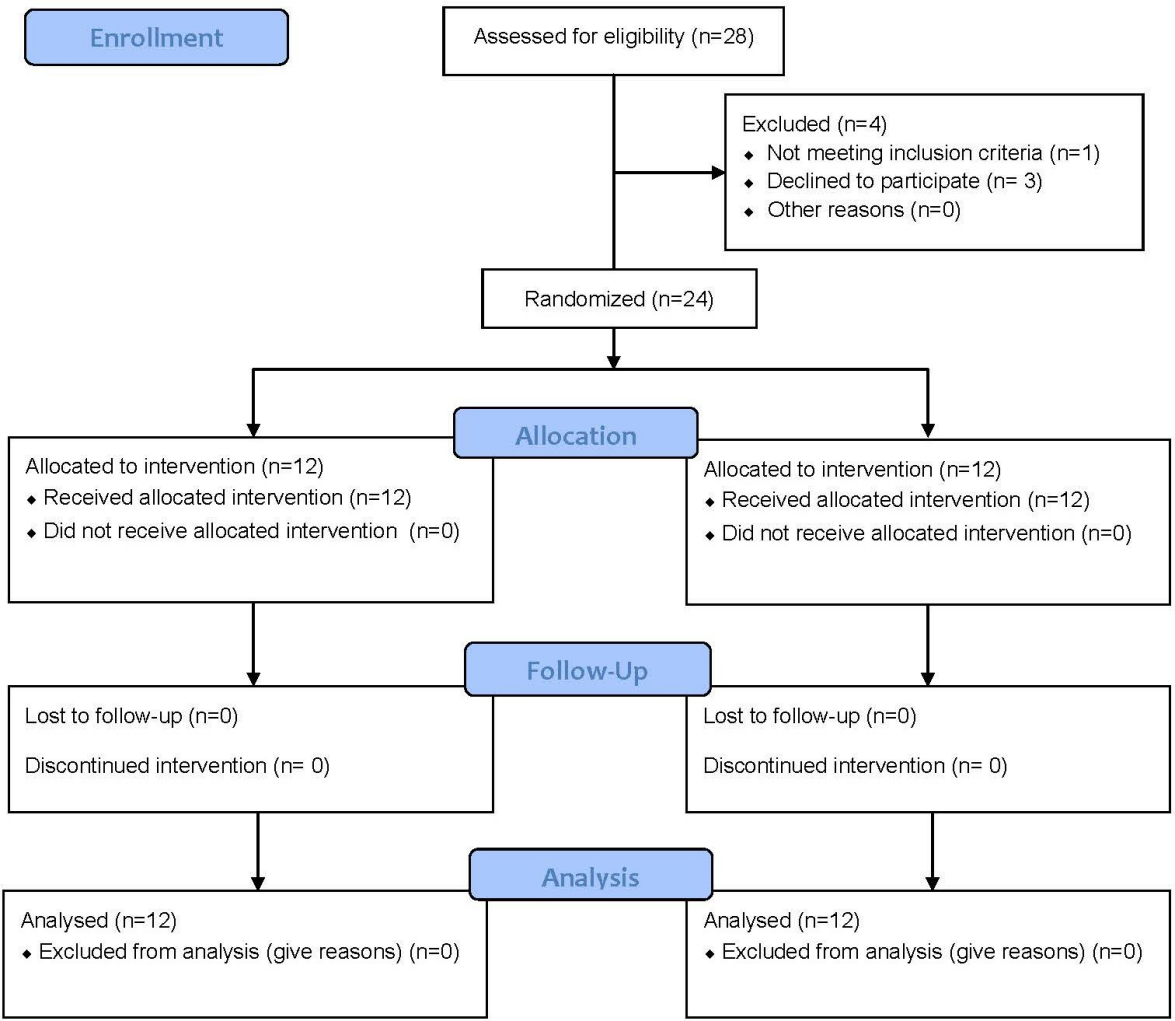
CONSORT participant flow chart.

To be eligible for this study, participant must be physically healthy with no injuries six months prior to the enrollment, and currently an active team member participating in daily training activity of the China women’s team. In addition, participant must be right-handed. Participants were recruited and tested between March to May, 2019 during pre-season when there was no competition. All eligible members of the China women’s team were inquired about their willingness to participate in this study, and those who agreed were enrolled in the study. A total of twenty-four female table tennis players from the Chinese women’s table tennis team participated in this study (Fig 1). This study used parallel design, and participants were randomly assigned into either INT intervention group or control group (CON) with 1:1 allocation ratio. The participants were each assigned with an integral number ranging from 1–24 first as their ID, and a custom-written Matlab (Version 2018, The Mathworks, Natick, MA, USA) script was used to determine the allocation (INT or CON group) of the number so that the participants were randomly assigned into each group. Prior to any data collection, participants were informed of all the testing and intervention procedures and provided written consent. Physical characteristics of all participants are summarized in Table 1.

**Table 1:** Participant demographic characteristics in the INT group (INT) and the control group (CG) (mean ± SD).

	INT (n = 12)	CG (n = 12)	p-value
Age (years)	23.5 ± 2.1	22.9 ± 2.4	0.430
Training experience (years)	10.4 ± 2.0	9.9 ± 2.7	0.881
Height (cm)	164.2 ± 4.5	163.0 ± 4.2	0.516
Body mass (kg)	58.7 ± 4.1	60.9 ± 8.1	0.504
BMI (kg/m^2^)	23.8 ± 4.1	24.5 ± 5.6	0.754

### Overview of procedures

All testing and intervention occurred at China Institute of Sport Science (Beijing, China). The intervention consisted of 4 training sessions (Monday, Tuesday, Thursday and Friday) every week for 8 weeks, with each session lasted about 30 minutes. Therefore, a total of 32 training sessions were performed. All training sessions were carried out in the morning after warming up and before their regular training. For each training session, INT group performed INT while CON performed normal physical training regime. Both INT and CON intervention protocols changed progressively from the first two weeks to week 3–5, and then changed again for week 6–8. Details of the training protocols are listed supporting document S1 Appendix. Both groups were tested before and after the 8-week intervention. The content of testing included 30-meter sprinting, 1 repetition maximal (1RM), vertical jump and Y balance test. These are all primary outcomes of the study.

### Testing protocols

The same research evaluated the same test for both INT and CON groups before and after the intervention. Strength was assessed using 1RM test for all participants. The test started with a 3-minute light weight warm-up. Afterwards, the participant performed squats starting with a predetermined weight which is known to be lower than her 1RM. Bilateral weights with a total weight ranging from 1–10 kg were added based on the participant’s report. A two-minute rest was given to the participant between two consecutive trials. The final weight that the participant was capable of enabling only 1 repetition was considered as 1RM. All participants reached their 1RM with 3–5 trials.

Mechanical power was assessed using vertical jump test and a Kistler (Kistler 9260AA, Switzerland) force plate. The participant stood on the force plate and performed 3 vertical jump tests using countermovement jump technique. Specifically, the test started with the participant stood straight on the force plate with both hands on the waist, then quickly squat down to a self-selected depth and then jump up as high as possible. The participant kept her hands on the waist throughout the test. A 30-second rest was given between two consecutive trials. The best result of the 3 trials was determined as the participant’s vertical jump test result. Prior to the data collection, a research assistant instructed the participant about the test, and 3–5 attempts were made by the participant to familiarize herself to the test. A minimum of 2-minute rest period was given between familiarization jumps and actual data collection.

Balance was assessed using Y balance test. The participant stood barefoot on the Y balance board (Y Balance Test KitTM, Danville, VA) with both hands on placed on the waist throughout the test. This was done to avoid the influence of upper limb swinging on the test results. The participant then bent her support leg to squat down, and the tiptoe of the testing leg tried to reach as far as possible on three directions, namely forward, posteromedial, posterolateral directions along the Y balance board. The participant was instructed to look forward during the test; if the testing leg touches the ground, or the blocks were kicked, or the participant loses her balance, then this trial was considered a failure and a re-test was carried out. The test score on each direction was calculated as the distance reached on that direction divided by leg length then multiply by 100. Each direction was tested twice, with a 1-minute rest given between different directions and trials. The longer distance achieved of the two trials was deemed as the Y balance test performance on that direction.

Speed was assessed using a 30-meter sprinting test and a straight track was used. A 30-meter distance was measured out with markers to indicate the starting and finishing lines. During the test, participants prepared herself at the starting line, and a research assistant who was a professional track coach with many years of experience whistled to signal the start of the test and used a stopwatch to record time to the nearest 0.01 second at the finishing line. A second research stood at the starting line to ensure that the participant start the test properly. Each participant did two trials with a minimum of two-minute rest given in between, and more rest time was given if the participant request. The shorter time was chosen to represent 30-meter trial performance.

### Data analysis

Based on previous studies that investigated the effects of INT intervention in athletes that included testing components similar to our study (strength, power/jump performance, speed and balance), the estimated effect size for our study was set to 0.7. Sample size estimation was calculated using G*power software [19] with alpha level set to 0.05 and power set to 0.8. Using ANCOVA test and an effect size of 0.7, it was calculated that a total of 18 participants were required for our study. Considering the potential loss of participants due to various reasons as well as the number of participants eligible for our study, a total of 24 participants were enrolled in our study.

Results are presented as Mean ± standard deviation. Normality was checked for all data prior to conducting statistical analyses and all data were normally distributed. Accordingly, independent t-test was used to determine whether significant differences exist for demographic characteristics between INT and CON groups (Table 1). ANCOVA was carried out with group as the between-subject factor, time (before and after intervention) as the repeated factor, and baseline measurement as a to test out whether training influences 30-meter sprinting, 1RM, vertical jump and Y balance test results. Post-hoc analysis using one-way repeated ANOVA and controlled for baseline measurement was performed if a significant interaction was detected. All statistical analyses were carried out using SPSS (version 24 IBM, Armonk, NY). To evaluate effect size, Cohen’s d (d) was calculated. Alpha level was set at 0.05.

## Results

Twelve participants were assigned to the INT group, and 12 were assigned to the CON group. All participants completed all the training as well as testing sessions, with no adverse events reported. Therefore, the retention rate was 100% in this study. No between groups differences were found for any test parameters prior to intervention (all *p* > 0.05). For 1RM test, there was a significant interaction effect (*p* < 0.001); post-hoc analysis showed that INT group increased 1RM by 11.6% after training (*p* < 0.001), while no difference was found for CON group (*p* = 0.069; Table 2). For vertical jump test, there was a significant interaction effect (*p* < 0.001); post-hoc analysis showed that INT group increased vertical jump height by 15.4% after training (*p* < 0.001), while no difference was found for CON group (*p* = 0.091; Table 2).

**Table 2:** Pretest and posttest results for physical performance following 8 weeks of intervention in INT group (INT, n = 12) and the control group (CG, n = 12).

	Group	Pretest	Posttest	%change	d	P-value for interaction
Sprint 30-m (s)	INT	4.72 ± 0.29	4.61 ± 0.28	-2.29%	0.392	0.122
	CG	4.70 ± 0.34	4.69 ± 0.32	-0.16%	0.032	
1-RM (kg)	INT	100.41 ± 9.88	112.08 ± 11.37^†^	11.61%	1.144	< 0.001*
	CG	100.83 ± 12.40	102.08 ± 11.57	1.24%	0.108	
Vertical Jump (cm)	INT	31.33 ± 3.41	36.16 ± 4.25^†^	15.41%	1.308	< 0.001*
	CG	31.44 ± 4.07	32.31 ± 4.16	2.68%	0.219	

d, effect size measured by Cohen’s d.

*significant interaction

^†^significant difference from baseline.

**Fig 2:**
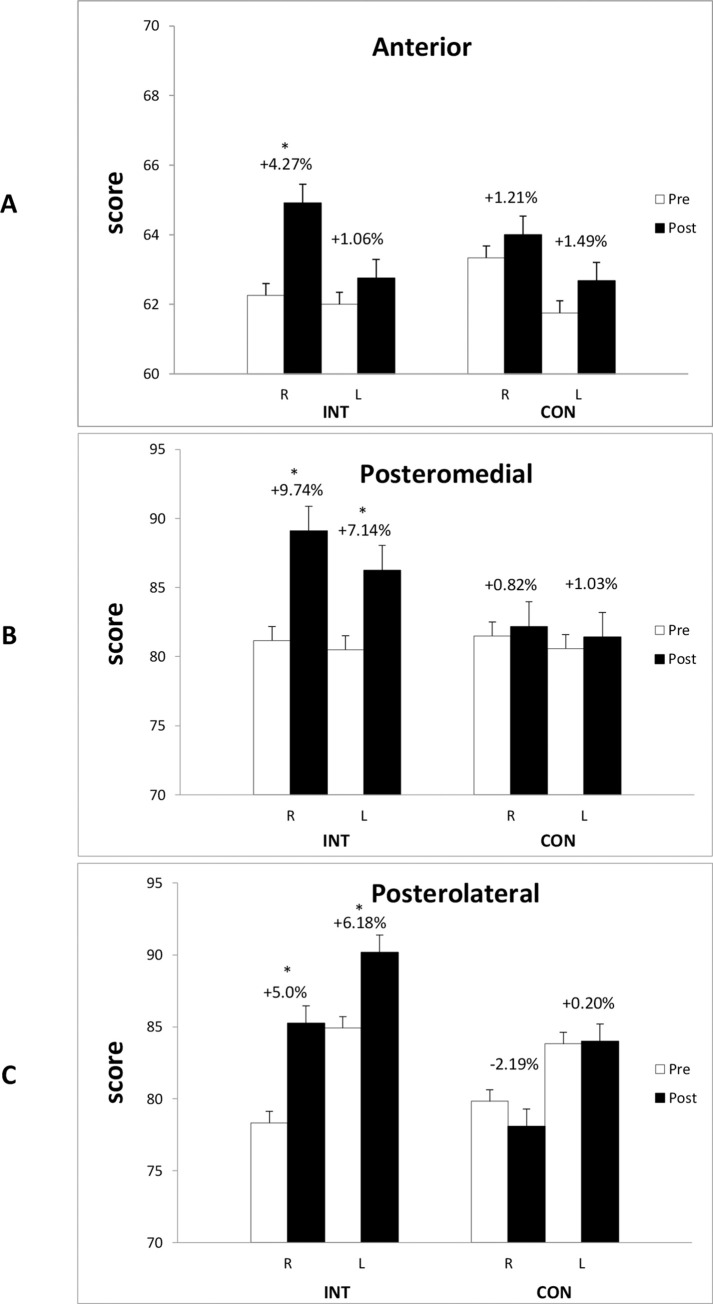
Y balance test results. INT, integrative neuromuscular training group; CON, control group. * Significant difference between baseline and post training.

For Y balance test, no significant interaction effect was found at the anterior direction for the left leg (*p* = 0.961). However, there was a significant interaction for the right leg at the anterior direction (*p* = 0.028). Post-hoc analysis revealed that INT group significantly increased reaching distance by 4.27% (*p* = 0.003; Fig 2A), and no changes were detected for the CON group (*p* = 0.203). Significant interaction effects were detected for posteromedial direction for both legs (both *p* < 0.05). Post-hoc analysis showed that INT group increased distance by 9.74% and 7.14% for right and left leg (both *p* < 0.05; Fig 2B and 2C), respectively, while no changes were found for either leg for CON group (both *p* > 0.05). In addition, there were significant interaction effects for posterolateral direction for both legs (both *p* < 0.05). Post-hoc analysis showed that INT group increased distance by 8.83% and 6.18% for right and left leg (both *p* < 0.05), respectively, while no changes were found for either leg for CON group (both *p* > 0.05).

For 30-meter sprinting test, there was no significant interaction effect (*p* = 0.122; Table 2).

## Discussion

To our knowledge, this is the first study that examined the effects of INT on physical performance in professional female table tennis players. The results of the current study partially support our hypothesis that INT can improve strength, power and balance in athletes as shown by the improvement in 1RM, vertical jump height and Y balance test. However, no significant improvement was seen for speed during sprinting following INT training.

INT is an emerging training modality, which integrates many training components together. Depends on the specific purpose, an INT protocol could include strength, power, agility, dynamic stability and coordination training. Compared to traditional training methods which usually emphasize on one or several limited physical fitness aspects, INT training has basic components of strength and speed exercises, while simultaneously focuses on dynamic stability, coordination and proprioception. As a result, it is generally considered that INT intervention is beneficial for physical fitness not in a specific but more comprehensive way, which is demonstrated by studies showing multiple physical performance improvements following training [20, 21]. Moreover, due to the integrative nature of this training modality, it enhances physical fitness in a time-efficient manner. The effectiveness, comprehensiveness and efficient characteristics of INT have led it being adopted by many sports teams as part of their fitness training routine. Most previous studies using INT intervention have focused on either youth or non-professional athletes [16, 21, 22]. Here we sought to evaluate the efficacy of INT intervention for professional female table tennis players. The results are encouraging, as they showed that despite the high physical fitness level already possessed by the players, the INT protocol we designed successfully enhanced most aspects of their physical fitness. Our study reassured that INT can be useful in training even the most elite professional athletes. However, how the improvement in physical fitness resulted from INT can be translated into sports/occupational performance is unknown and requires further investigation.

The squat and weight-carrying squat exercise in our INT protocol were designed based on athlete’s 1RM [23, 24], with the purpose to increase strength of major muscle groups. Our data suggest INT is effective in improving maximal strength, as shown by the results of 1RM which increased by 11.67 kg (11.61%) in INT group following intervention. This finding is consistent with other studies which concluded that INT is effective in improving strength in elite athletes [20, 25]. Strength gain is important for sports performance in table tennis players in many ways. For example, research indicated that a stroke is a chain of movements starting from the lower extremities, which eventually combine forces from the whole body together [10]. Different levels of players show significant differences on the quality of the stroke movement, and those with better strength and power tend to complete the stroke with better quality [8]. Therefore, the improved strength after INT intervention is likely translated into better stroke quality for the players, although more research is needed to confirm this inference.

In this study, INT group increased vertical jump height by 15.4%, which is consistent with a study showing significant jump height increase in female college players after 6 weeks INT intervention [26], while no significant changes were observed for CON. Lower extremity exercise performance, such as jump height, is related to both muscle strength and power [27, 28]. In our study, the INT protocol incorporated a large component of muscle training, both for the whole body (such as squat) as well as specific lower extremity muscles (such as prone leg curl). This will likely yield lower extremity muscle strength and power improvement, which is partly reflect by the increased 1RM results following training. Moreover, the INT protocol we adopted is consisted of many variations of jump exercise. These are designed to improve lower extremity power for the athletes; however, they may provide task-specific benefits other than just power and strength, although more research is needed. Taken together, it is likely that the increased jump height in the INT group is the result of the combined benefits from the multi-facet training regime of the intervention protocol. Lower extremity strength and power are crucial for table tennis players. Table tennis is a sport characterized with abrupt blocking movements [29], which means sudden termination and initialization of movement is common. Those with good lower extremity strength and power will likely perform these movements with better quality, which translates into better sports performance and lower injury risks, although further studies are needed to confirm this notion.

The results of Y balance test suggested that INT group improved at both posteromedial and posterolateral directions, and right leg at the anterior direction, which are consistent with previous studies that found INT intervention can improve balance performance in athletes [22, 30]. Specifically, to promote balance for our participants, the INT protocol was designed to include a core muscle strength training component, which in theory should yield strength increment in lumbar and spinal muscles and hence increase core stability. On the other hand, the lower extremity strength training component could increase hamstring strength in the INT group, which is known to be weak in female athletes [31] but crucial for knee joint stability [32]. The resulted knee joint stability enhancement could be one of the mechanisms for the improved Y balance performance in the INT group. In addition, the INT protocol did include multiple movements to various directions, which can increase peripheral and central nervous system adaptation, hence increase the proprioception of the lower joints [33]. Therefore, training components of core muscle strength, hamstring muscle strength and proprioception likely yielded improvement for the respective factors, and the combined effect is presented as the increased Y balance scores. Interestingly, INT group improved balance at the anterior direction on the right leg but not the left leg. It’s unknown why this discrepancy exists; however, considering that all the participants in the current study use right hand grip, it’s possible that this improvement may have positive implications for sports performance in these athletes. Whether the side-specific balance improvement is related to their side-dominance, and whether the overall improvement of balance could be translated into stability for sports performance in table tennis player warrants further investigation.

The results of the 30-meter sprinting test are inconsistent with our hypothesis that INT intervention can increase speed in female table tennis players. Specifically, neither INT group nor CON group showed improvement post training compared to baseline (both *p* > 0.05). This is contrary to previous studies which showed INT intervention can improve sprinting speed [21]. One possible explanation for this discrepancy is the lack of relative training component, as speed was not a focus for our INT protocol. While speed is essential for table tennis players, sprinting for 30 meters may not reflect the proper sport-specific requirement for speed, as their movement distances for one round of stroke is usually limited and more emphasize on the initialization and termination phase, as we discussed before. On the other hand, all subjects in the current study are elite athletes with very high physical fitness levels. It’s possible that there may be a ceiling effect for our participants, which means that there is limited room for improvement to begin with. These factors may explain the lack of sufficient improvement in sprinting performance following INT intervention in this study.

Interestingly, in this study we found that INT can significantly improve jump performance but not sprinting performance in our participants. It has been documented that sprinting capacity is significantly correlated with vertical jump height in athletes [34–37], which seems to be contradictory to our findings. The discrepancy could be attributed to several reasons. First, previous studies have primarily focused on soccer and basketball players, and the training content in these studies put a great emphasis on short-distance sprinting training due to the characteristics of the sport, which was not the case for table tennis players. The relatively limited sprinting-oriented training content in our INT protocol may partially explain the dichotomous findings in our study. Second, although not significant, we did observe a trend towards increased sprinting speed in the INT group with moderate effect size following training (*d* = 0.392), while the CON group showed virtually no change at all (*d* = 0.032). It is therefore reasonable to postulate that INT intervention could also promote sprinting performance in table tennis players, only to a lesser degree than jump performance, although more research with larger sample sizes and/or longer training period are needed to confirm this inference. Taken together, it seems that although INT intervention can potentially promote sprinting and jump performance simultaneously, specific training content targeting each element may be required to achieve such goal.

There are limitations associated with this study. First, it is well known that upper limb muscles are essential for the performance of racket sports such as tennis and table tennis [38, 39], therefore information on how training intervention may influence upper limb muscle strength and power could be informative for those who practice racket sports. However, in this study we didn’t assess upper limb muscle strength or performance. The lack of specific measurements to test the effects of INT on upper limbs is a limitation of the current study and should be addressed in future studies. In addition, current study did not assess whether INT intervention can be used to prevent injury or whether the benefits from INT intervention can be translated into better longitudinal health outcomes in athletes. Future longitudinal studies with these specific purposes are needed.

## Conclusion

The results of the current study suggest that an 8-week INT intervention can significantly improve physical performance in athletes. The improvements seen in 1RM, vertical jump and Y balance test in INT group demonstrated the feasibility of INT as an integrative training modality for professional table tennis players. INT could be integrated as a routine physical training modality to enhance physical performance and promote sports performance in table tennis players.

## Supporting information

S1 Checklist CONSORT checklist. (DOC)

S1 File Study protocol in Chinese. (DOCX)

S2 File Study protocol in English. (DOCX)

S1 Appendix Detailed training protocol. (DOCX)
