# Supplementary material for: Effects of integrative neuromuscular training intervention on physical performance in elite female table tennis players: A randomized controlled trial
Source: PLoS One. 2022 Jan 20;17(1):e0262775. doi: 10.1371/journal.pone.0262775 (PMC8775216; doi:10.1371/journal.pone.0262775)
Supplement: S1 File — (DOCX) [file pone.0262775.s002.docx]

**研究计划书**

**研究题目**: 整合性神经肌肉训练对女子职业乒乓球运动员体能表现的研究

**项目号**: CISSIRD-20190104

**主申请者信息**:

肖丹丹，国家体育总局体育科学研究所运动心理学及生物力学研究中心. 电子邮件: [xiaodandan@ciss.cn](mailto:xiaodandan@ciss.cn). 电话: +8613911307737

**其他申请人及所属单位**:

熊金凤 国家体育总局体育科学研究所运动心理学及生物力学研究中心

李上校 国家体育总局体育科学研究所运动心理学及生物力学研究中心

钱磊 西安工业大学

曹爱斌 山西大学

彭博 中国政法大学

**1. 研究目的**:

这项研究主要目的是探讨整合性神经肌肉训练能否提高职业女子乒乓球运动员的肌肉力量，加快运动员速度，改善平衡能力。虽然以往研究表明，整合性神经肌肉训练可以提高儿童身体表现能力，但对职业运动员的研究并不多，针对乒乓球运动员的研究目前还没有。

**2. 研究类型**:

本研究为随机对照实验。

**3. 研究地点**:

国家体育总局体育科学研究所运动心理学及生物力学研究中心。

**4. 资助单位及项目号**:

本研究由中国国家体育总局业务基金(基金号20-07), 以及中国政法大学(基金号1181/23320055)资助.

**5**. **研究背景**:

本研究旨在确定整合性神经肌肉训练这一创新训练模式在女子职业乒乓球运动员中的应用，重点为确定该训练模式是否可以提高运动员的力量，速度和平衡素质。前期研究证实该训练模式可以同时提高运动员的多种身体素质，然而针对职业运动员的研究较少，针对乒乓球运动员的目前还不存在。因此本研究有重要的实际意义，可以弥补相关文献的空缺。

**6. 受试者纳入及排除条件**：

纳入条件包括1) 现役国家女队乒乓球运动员, 2) 身体健康，无伤病, 3) 右手持拍。 排除条件包括: 1) 过去六个月中曾受伤，主要是骨骼肌肉类伤病, 2) 不愿参加本项研究。

**7. 研究步骤**:

**7.1 研究总体过程**

符合纳入条件的中国国家女子乒乓球队运动员将被随机分配到两个训练组，一组为整合性神经肌肉训练组，另一组为对照组。两组均会进行训练前及训练后的两次测试，测试内容完全相同。所有运动员进行为期8周的干预训练，每周4日（周一、周二、周四、周五），共32次，每次持续时间30分钟。训练后的测试的时间在最后一次训练完成后的两天内。

**7.2 测试步骤**。

**7.2.1** 身高及体重将分别使用身高和体重计进行测量。

**7.2.2** 在所有测试开始前，受试者将在专业教练员带领下进行10分钟左右的常规热身活动。在受试者的身体充分热身、拉伸后将进行第一项测试--垂直纵跳测试，这项测试在Kistler（Kistler 9260AA，Switzerland））测力台进行。受试者将脱去鞋子、袜子，双脚站在测力台上，手臂弯曲置于躯干两侧，每次均尽力往上跳跃。受试者将进行三次测试，每次测试间隔至少30秒，选择最好测试成绩作为样本。

**7.2.3** 受试者来到体能训练中心30米跑道进行测试，跑道的起点和终点都有标志物，一名研究助理在起跑处吹哨，受试者听到口令时全力跑向终点，另一名助理研究员使用秒表，记录测试成绩，受试者将会有两次测试机会，选择最好成绩作为研究样本。

**7.2.4** 受试者将进行Y平衡测试。在测试之前研究助理会向受试者解释测试工具使用方法，播放视频演示标准化测试动作，受试者可以向研究助理提出受试者的疑问，在无任何疑问后，开始测试。受试者一只脚站在Y平衡测试仪中间脚板（Y Balance Test KitTM，Danville，VA）上，为避免上肢摆动对测试动作的影响，在测试过程中受试者需要保持双手叉腰。支撑腿屈膝下蹲，测试腿的脚尖分别向前、后内、后外沿着Y平衡测试套件的三个方向作最大努力移动，直至达到最大距离。整个测试过程要求受试者正视前方，如果脚触地、踢动挡板、躯体失去平衡视为失败。动态姿势控制能力测试指标结果为：3个方向得分/腿长×100。每次测试动作之间休息1分钟，每个方向测试两次，选择最好成绩纳入样本。

**7.2.5** 受试者将进行1RM（一次性最大肌肉力量）测试。首先专业教练员会告知受试者之前力量训练的情况，受试者和教练员共同确定一个开始负荷。接下来，受试者将穿戴好护具，在测试前完成3分钟的轻负荷热身。我们会有两名研究助理站在杠铃架两侧对受试者进行测试保护，同时也方便为受试者增加两侧杠铃片，增加的范围为1～10公斤，在3～5次尝试达到1RM，每次尝试之间休息2分钟。最终确定的重量为1RM结果。

**7.3 随机分配过程**。

所有受试者将会被分配一个ID号码，该号码将被用作随机分配组别。随机分配将由我们自主开发的Matlab程序完成。

**7.4 训练干预。**

**7.4.1** 如果受试者被分配至试验组，我们将会对受试者实施8周体能训练干预，每周一、周二、周四、周五，在受试者日常训练结束之后，我们的专业教练员会指导受试者参与整合性神经肌肉训练，持续时间为30分钟。干预训练时间为8周，共分为三个阶段：1-2周为低难度训练，负荷强度一般；3-5周为中高难度训练，负荷强度大；6-8周为高难度训练，负荷强度大。整个干预过程，训练内容难度从低难度过渡到高难度。

**7.4.2** 如果受试者被分配至对照组，那么受试者在完成第一次测试之后，在接下来8周训练中，受试者也是在每周一、周二、周四、周五，在受试者日常训练结束之后，进行常规体能训练。在与受试者的教练员沟通后，确定训练内容为受试者之前最常用的内容，训练持续时间同样为30分钟。

**8. 风险和利益**:

**8.1** 风险和应对措施

**8.1.1** 受试者应当在在测试之前检查自己的运动装备，避免因为运动鞋不合脚等原因出现摔倒等意外。受试者在到达30米终点后，不能立即停止运动，需按照要求减速慢跑直至心率逐渐恢复，避免短时间剧烈运动导致头晕或恶心等不适。

**8.1.2** 受试者可能会在测试后感到肌肉酸痛，它将在测试2天后缓解；迟发的或不能缓解的肌肉肿胀、疼痛，我们会尽快联系在场研究助理采取进一步处理。

**8.1.3** 在测力台上进行光脚垂直纵跳，受试者的足部可能会因为没有鞋子的缓冲而出现不适，这种情况在停止测试5分钟后将会消失，如果有持续疼痛不能缓解，我们同样会尽快联系在场的研究助理采取进一步处理。

**8.1.4** 受试者在单脚支撑下完成相关动作，有可能腿在前伸过程中出现腿部或是臀部肌肉拉伤。此外，可能测试时间过长或由于躯干、下肢支撑力量不平衡，出现摔倒。因此，研究助理会根据受试者的当时情况，采取适当的保护措施，以减少跌倒风险。

**8.1.5** 运动干预可能会导致受试者有不适，肌肉酸痛等症状，这些症状应当在休息不久后就可以适当缓解。如果症状持续较长，我们有队医可以帮助治疗。跌倒及肌肉拉伤也是干预可能导致的意外事件。为降低这些状况发生的可能性，我们所有的训练干预都会有体适能教练在场观察。如受试者感觉不能继续进行干预，可自行停止。

**8.2 利益**

**8.2.1** 训练可能会在速度、力量、弹跳和平衡能力等方面得到改善。在研究结束时，受试者将获得自己的肌肉力量、身体充实度（如上臂围、大腿围）、平衡和身体活动水平的信息。

**9. 受试者报酬**

每完成一次测试，受试者将获得100元测试费用津贴。在为期8周的训练时间内，受试者每天完成训练后，将会获得20元津贴，完成4周训练任务将会获得300元津贴，完成8周训练任务，受试者将会收到640元津贴。

**10. 受试者个人信息保密**

未经受试者的书面许可，受试者的个人身份信息、个人记录、问卷和测试结果，将不会与其他不参与本项目的人共享。与受试者有关的问卷、测试结果都采用数字编号，因此本研究的工作人员和审查人员（伦理审查委员会）只能看到记录的数字编码，而不能看到受试者的姓名，以后发表的研究结果也不包括受试者的个人信息。该项目的研究记录可能由国家体育总局体育科学研究所负责监管和监督的部门审查。
