# Supplementary material for: Effects of integrative neuromuscular training intervention on physical performance in elite female table tennis players: A randomized controlled trial
Source: PLoS One. 2022 Jan 20;17(1):e0262775. doi: 10.1371/journal.pone.0262775 (PMC8775216; doi:10.1371/journal.pone.0262775)
Supplement: S2 File — (DOCX) [file pone.0262775.s003.docx]

**Study Protocol**

**Study title**: Effects of integrative neuromuscular training intervention on physical performance in elite female table tennis players: a randomized controlled trial

**Study No**: CISSIRD-20190104

**Applicant information**:

Dandan Xiao, Research Center for Sports Psychology and Biomechanics, China Institute of Sport Science. Email: [xiaodandan@ciss.cn](mailto:xiaodandan@ciss.cn). Phone: +8613911307737

**Other investigators and affiliations (list all)**:

Jinfeng Xiong, Research Center for Sports Psychology and Biomechanics, China Institute of Sport Science.

Shangxiao Li, Research Center for Sports Psychology and Biomechanics, China Institute of Sport Science.

Aibin Cao, School of Physical Education, Shanxi University.

Lei Qian, School of Sciences, Xi’an Technological University.

Bo Peng, Department of Sports, China University of Political Science and Law.

**1. Purpose of the project**:

The purpose of this project is to determine the effect of integrative neuromuscular training (INT) on physical performance in professional table tennis players. Specifically, we will examine how their muscle strength, speed and balance change following training. Previous studies have investigated the effect of INT on professional athletes; however, relative studies are scarce, and studies examining how INT may have an effect on table tennis players has yet to be conducted.

**2. The type of research project**:

This is a randomized controlled trial.

**3. The location where the project is going to be conducted**:

All procedures will be conducted at the Research Center for Sports Psychology and Biomechanics, China Institute of Sport Science.

**4. Funding agency and grant number**:

This research is funded by the fundamental research funds of China Institute of Sport Science (grant No. 20-07), and the China University of Political Science and Law (grant No. 1181/23320055).

**5**. **Background**:

This study aims to determine how INT, a novel strength training procedure may influence the muscle strength, speed and balance aspects of physical performance in professional table tennis players. Previous studies indicated multiple aspects of physical performance gain for those who performed INT; however, studies the examined how INT may improve physical performance in elite athletes, a population who already possesses high level of physical skills, are scarce. Filling this knowledge gap could help researchers determine whether INT is a useful training paradigm for professional athletes.

**6. Inclusion/Exclusion criteria**：

Inclusion criteria include1) female professional player currently enrolled in the Table Tennis National team, 2) physically healthy without any injuries, 3) use right hand as the play side. Exclusion criteria include : 1) musculoskeletal injury within the past six months, 2) unwilling to participate in the study.

**7. Study procedures**:

**7.1 Overall procedures**.

Eligible participants will be recruited from the Chinese Women’s Table Tennis team and will be randomized into either INT group or control (CON) group. Both groups will be tested at baseline and after the intervention with details described below. Testing procedures are the same for all participants. After baseline testing, participants go through 8-weeks training protocols, with specific training contents vary based on which group they are assigned to. Training occurs every Monday, Tuesday, Thursday and Friday as part of the physical training routine. All training sessions are about 30 minutes. Two days after the last training session, post-intervention testing will be performed on all participants.

**7.2 Testing procedures**.

**7.2.1** Height and weight will be measured using a stadiometer and weight scale, respectively.

**7.2.2** Jump height will be determined using a Kistler (Kistler 9260AA, Switzerland) force plate. After a 10-minute warm up period consisted of jogging and stretching, participant will remove her shoes and socks, and then stand on the force plate with hands on her waist. After the test starts, the participant will jump as high as possible for three times, with at least 30s resting time in between. The highest height achieved will be used as the jump height.

**7.2.3** Speed will be determined on a 30-meter racetrack. Participant prepares herself at the beginning line, and a research assistant will blow the whistle to indicate the start of the test. The participant will be encouraged to run as fast as possible, with running time being recorded by the research assistant using a stopwatch. The participant will be tested twice with sufficient resting time in between. The better trial will be used to indicate speed.

**7.2.4** A research assistant will show the participant how the Y-balance test is performed, and answer any questions the participant may have. The participant will then stand barefoot on the Y balance board (Y Balance Test KitTM, Danville, VA) with both hands on placed on the waist throughout the test. The participant will then bent her support leg to squat down, and the tiptoe of the testing leg try to reach as far as possible on three directions, namely forward, posteromedial, posterolateral directions along the Y balance board. The participant will be instructed to look forward during the test; if the testing leg touches the ground, or the blocks are kicked, or the participant loses her balance, then this trial is considered a failure and a re-test will be carried out. The test score on each direction will be calculated as the distance reached on that direction divided by leg length then multiply by 100. Each direction will be tested twice, with a 1-minute rest given between different directions and trials. The longer distance achieved of the two trials will be deemed as the Y balance test performance on that direction.

**7.2.5** One-repetition maximal (1RM) test will be performed to determine the maximal strength of the participant. The test be followed by a 3-minute warm-up period. Afterwards, the participant wears protective gear and start the test with a load that’s known to be tolerable to her. Two research assistance will stand on each side of the rack to protect the participant. Weights will be added bilaterally for a total of 1-10 kg every time, based on participant’s response, with 2 minutes reach given in between. 1RM should be reach within 3-5 trials. The final weight will be deemed as 1RM.

**7.3 Randomization**.

The participants will each be assigned with an integral number first, and a custom-written Matlab script was used to determine the allocation of the number.

**7.4 Intervention procedures**.

**7.4.1** INT group will perform INT training every Monday, Tuesday, Thursday and Friday for 30 minutes throughout an 8-week period. Training will be supervised by professional strength coaches. The INT intervention will be divided into three stages: week 1-2 will be low load/intensity training period; week 3-5 will be moderate load/intensity training period; and week 6-8 will be high load/intensity training period. Specific training contents is listed separately.

**7.4.2** Control group will perform regular physical training every Monday, Tuesday, Thursday and Friday for 30 minutes throughout an 8-week period. Training will be supervised by professional strength coaches. Training content will be similar to their previous physical training regime, with specific training details listed separately.

**8. Risk and benefits**:

**8.1 Risks**

**8.1.1** Sprinting as fast as one can then stop may make one nauseated and want to throw up. And there is a slight chance of falling during sprinting. In order to avoid risks, the participant will be instructed to check her gear (shoes) and continue running and slowly down after reaching the destination. A research assistant will check the track for abnormal object presence every time before the participant sprints.

**8.1.2** Muscle soreness is a common side effect for strength training, which could also happen after 1RM test. This is common and should go away in a couple days. If muscle soreness persists, the participant can ask a research participant for further assistance.

**8.1.3** Vertical jump test requires the participant to jump barefoot on a plate. Discomfort may occur due to the high load applied directly to the foot. This should go away within 5 minutes. If not, the participant can ask a research participant for further assistance.

**8.1.4** There is a slight chance that muscle strain injury may occur during Y balance test. In addition, falls may occur when the participant cannot maintain her balance. Mattress will be put around the participant to minimize the risk of falling. Research participant will be around to help the participant to minimize risk of muscle strain injury and falling.

**8.1.5** Discomfort, muscle soreness may occur during training intervention, which should all go away soon. If not, we have team physician on call to help. Fall and muscle strain injuries are also possible during intervention. All training sessions are supervised by professional strength coaches and if there are signs of injury, training will be stopped. If the participant feels the need to stop, she can request to do so.

**8.2 Benefits**

**8.2.1** Participant may have strength, speed balance and power gain following the intervention. We will provide them a report once all procedures are finished.

**9. Incentives**

To facilitate recruitment and retention, 100 yuan will be provided to participants once they complete a testing session. In addition, 20 yuan will be provided to participants to participants upon completing a training session. Therefore, the participant could get 200 yuan for completing both baseline and post-intervention training sessions, and 640 yuan for completing all training sessions.

**10. Confidentiality**

No individually identifiable information or any information provided by the participant will be shared with other s without the written permission of the participant. Each participant will be assigned with an ID number that will be used for all study records. The only record that can link the ID number with the specific participant will be locked in a secure cabinet where the PI has the sole access. The participant’s research record may be reviewed by the Institutional Review Board at China Institute of Sport Science.
