# Supplementary material for: Effects of integrative neuromuscular training intervention on physical performance in elite female table tennis players: A randomized controlled trial
Source: PLoS One. 2022 Jan 20;17(1):e0262775. doi: 10.1371/journal.pone.0262775 (PMC8775216; doi:10.1371/journal.pone.0262775)
Supplement: S1 Appendix — (DOCX) [file pone.0262775.s004.docx]

Appendix A. Integrative neuromuscular training (INT) and control group (CG) training protocols.
